# Intravascular Large B‐Cell Lymphoma Presenting as Recurrent Cryptogenic Strokes: A Diagnostic Challenge

**DOI:** 10.1155/crh/9959762

**Published:** 2026-09-18

**Authors:** Veena Krishnan, Habtemariam Dessie, Kimberly Stogner, Jakita Baldwin, Roy Strowd, Rakhee Vaidya, Sarada Krishnamurthy

**Affiliations:** ^1^ Department of Internal Medicine, Advocate Health Wake Forest Baptist, Winston-Salem, North Carolina, USA, unc.edu; ^2^ Department of Neurology, Advocate Health Wake Forest Baptist, Winston-Salem, North Carolina, USA, unc.edu; ^3^ Department of Pathology, Advocate Health Wake Forest Baptist, Winston-Salem, North Carolina, USA, unc.edu; ^4^ Department of Cancer Medicine, Advocate Health Wake Forest Baptist, Winston-Salem, North Carolina, USA, unc.edu

## Abstract

Intravascular large B‐cell lymphoma (IVLBCL) is a rare malignancy and is a challenging diagnosis to make given its varied clinical presentation. Here, we present the case of a 69‐year‐old male who presented with multiple cryptogenic strokes. Despite multiple hypercoagulable and malignancy workups, no etiology was identified, with multiple CT scans negative for any masses. Ultimately, a vascular abnormality was noted on a CTA of the head and neck, and IVLBCL was diagnosed on biopsy. The patient was initiated on R‐CHOP and had improvement in his neurological status after the initiation of chemotherapy, and his ClonoSeq MRD testing is negative. This case is of interest given the lack of imaging findings for this patient prior to diagnosis as all of his CT scans were negative, making it a challenge to diagnose him as well as monitor his response to treatment. Additionally, his case highlights the importance of ClonoSeq MRD testing, which allows for the detection of even minimal amounts of disease in tracking response to treatment.

## 1. Introduction

Intravascular large B‐cell lymphoma (IVLBCL) is a relatively rare entity with an incidence rate of 0.095 per 1,000,000 per year in the United States. Diagnosing this disease is complicated as clinical presentations can greatly vary and overlap with other disease states. There are three clinical variants of IVLBCL: the classical variant, the cutaneous variant, and the hemophagocytic variant. For the classical variant, most cases present with traditional constitutional symptoms, including fevers, weight loss, night sweats, and chills [1]. Cutaneous manifestations of the disease are also possible, appearing in about 40% of presentations, ranging from the peau d’orange rash of the breast to smaller plaque‐like lesions. For the hemophagocytic variant, there is typically evidence of bone marrow involvement and significant cytopenias [1]. Patients can also have symptoms based on which organ is involved. For instance, patients with CNS involvement can present with progressive encephalopathy, paraparesis, and strokes, often with increasing and evolving infarcts on imaging [2]. In fact, some patients with underlying IVLBCL are thought to have cryptogenic strokes as they have recurrent, worsening symptoms with no clear etiology until the malignancy is discovered [3]. Given this potential manifestation of IVLBCL, it is important to pursue a broad workup for cryptogenic stroke, given that treatment of the underlying lymphoma can significantly reduce symptom burden and can potentially lead to improvement in sensory and motor function.

## 2. Case Report

A 69‐year‐old male with a past medical history of SIADH, neurogenic claudication secondary to lumbar spondylosis, and prior L2–S1 posterior lumbar interbody fusion surgery originally presented to an outside facility with right lower extremity weakness and aphasia. Imaging revealed multiple small acute and subacute ischemic infarcts in the centrum semiovale bilaterally, with no large vessel occlusion. The patient was not a candidate for tPA or thrombectomy and was treated conservatively with aspirin and atorvastatin. He underwent TTE, TEE, and implantable loop recorder placement, which did not reveal any PFO, atrial shunting, or occult atrial fibrillation, respectively.

Three months later, he presented with further worsening of word‐finding difficulty and right lower extremity weakness. He was noted to have a small acute lacunar infarct in the left splenium of the corpus callosum and multifocal cortical restricted diffusion bilaterally. Repeat TTE and MRA of the head and neck were performed with no evidence of a PFO or any significant arterial stenosis. Given recurrent stroke‐like symptoms, the patient was initiated on apixaban 5 mg twice a day for a possible underlying hypercoagulable state.

Six months from the initial stroke‐like symptoms, the patient had recurrent worsening of right lower extremity weakness and word‐finding difficulty with a subsequent brain MRI that showed acute multifocal microinfarcts in the left superior frontal and parietal lobes. Repeat TTE was performed and was negative for evidence of a PFO. CTA of the head and neck demonstrated some opacification of the bilateral ACA arteries, without evidence of active occlusions. CT of the chest, abdomen, and pelvis was performed to rule out possible occult malignancy as a contributing cause for recurrent strokes. It revealed a nonspecific 2‐cm splenic lesion deemed appropriate for outpatient follow‐up.

Two weeks after his last hospitalization, the patient was admitted again for recrudescence of old stroke symptoms in the setting of fever and hypotension without evidence of new strokes on MRI. A PET‐CT was obtained to evaluate for underlying malignancy. The imaging was only notable for focal metabolic activity in the adrenal glands, thought to be secondary to active hyperplasia, with no evidence of active malignancy. Brain PET/CT was negative for perivascular hypermetabolic activity that would be suggestive of vasculitis, and the hypercoagulable workup was unrevealing.

**FIGURE 1:**
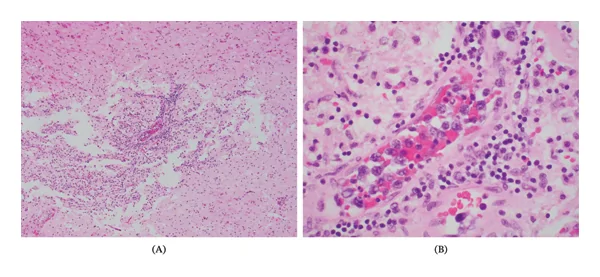
Intravascular malignant lymphocytes and perivascular small, non‐neoplastic lymphocytes are seen on H&E stains at 100X (A) and 600X (B).

**FIGURE 2:**
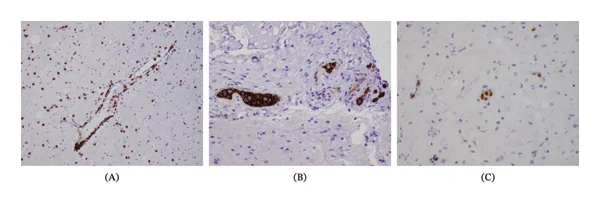
Immunohistochemical stains of the brain biopsy: (A) CD3 highlights perivascular T lymphocytes and is negative in the intravascular spaces. (B) CD20 and (C) MUM‐1 are positive in the malignant cells, which are confined to the intravascular spaces.

Shortly after discharge and 7 months from the initial onset of symptoms, the patient was brought to our facility for acute encephalopathy in the setting of hyponatremia. Anticoagulation was discontinued as the suspected diagnosis at that time was thought to be possible vasculitis or CNS malignancy. Cerebral catheter angiogram revealed nonspecific small vessel vasculitis, vasculopathy in all vascular distributions, and 50% stenosis in the bilateral ACA and MCA arteries proximally. While pending a brain biopsy for definitive diagnosis, the patient developed worsening aphasia and right‐hand weakness. Repeat brain MRI showed new, punctate, multifocal ischemic infarcts in the left frontal, right parietal, and right temporal lobes and bilateral cerebellum. CTA of the head and neck showed bilateral M2 and A2 stenosis along with a luminal irregularity, which were new compared to CTA images obtained 1 month prior. The patient was then started on high‐dose IV methylprednisolone with some improvement in symptoms. He then underwent a biopsy of the luminal irregularities in the brain, which revealed malignant B cells confined to the vascular lumina (Figure 1A,B; Figure 2B,C), consistent with IVLBCL. This site was selected because it represented the most radiographically active area of disease. Furthermore, PET‐CT, CT chest/abdomen/pelvis scans, and dermatologic examination failed to identify a more accessible site. A lumbar puncture was obtained, which was negative for any evidence of leptomeningeal disease.

As there were no clinically evident skin lesions, a skin biopsy was deferred. He was treated with R‐CHOP with symptomatic improvement and discharged to continue outpatient therapy. Currently, this patient has completed 6 cycles of R‐CHOP without intrathecal (IT) chemotherapy and has not had any worsening of his neurological deficits. Repeat MRI of the brain and spine did not demonstrate any progression of his disease, and ClonoSeq minimal residual disease (MRD) testing after completing 6 cycles of R‐CHOP was negative.

## 3. Discussion

As demonstrated in the case above, the diagnosis of IVLBCL is a challenging one to make as the presenting symptoms can overlap with other clinical syndromes [1]. Given the challenges in diagnosing the disease, it was often a postmortem diagnosis [4, 5]. Now, as the disease is more readily identified, prompt initiation of appropriate therapy leads to successful treatment [6]. Furthermore, even patients with extensive CNS involvement, which often portends a poor prognosis, can have sustained remission with the early initiation of chemotherapy [7]. One characteristic of IVLBCL that it shares with the other large B‐cell lymphomas is its responsiveness to R‐CHOP. The introduction of rituximab specifically represents an important milestone in treating IVLBCL with overall progression‐free survival at 2 years significantly improved. Given that the disease often has CNS involvement, CNS prophylaxis with IT chemotherapy is usually recommended if the patient has proven leptomeningeal disease [8]. In the case above, the patient had primary CNS disease, but given that his lumbar puncture was negative for any malignant cells, IT chemotherapy was deferred.

Our patient presented with multiple recurrent strokes despite optimized maximal medical therapy—a presentation that duly prompted a broad workup for underlying procoagulopathic states, inclusive of malignancy. Although CNS vasculitis was strongly considered in this patient, several laboratory features distinguish IVLBCL from CNS vasculitis. Typically, CSF analysis in IVLBCL patients will demonstrate relatively unremarkable findings, whereas CSF analysis in CNS vasculitis will typically demonstrate a lymphocytic pleocytosis. Additionally, primary CNS vasculitis does not typically have elevated systemic markers of inflammation such as ESR or CRP. IVLBCL can be associated with soluble IL‐2 receptor levels as well as positive circulating tumor DNA (ctDNA) [9].

Imaging modalities can be of great assistance, including MRI, CT, and PET scans. However, the findings are not always specific to IVLBCL and may not identify any evidence of disease [10]. Our patient had multiple workups for malignancy, though his complicated clinical course involved multiple repeated imaging—all negative for malignancy. In much of the literature on IVLBCL, these pan‐scans of the body typically reveal some type of lymph node or solid organ involvement, leading to a biopsy and subsequent diagnosis from those sites [11].

The lack of specific imaging findings in this case complicated early diagnosis and delayed treatment. The lack of imaging findings also posed a challenge in assessing treatment response, as typically imaging is the cornerstone for most disease states. Posttreatment brain and spine MRI revealed no evidence of disease, but there was no spinal involvement even at the time of diagnosis. As such, much of assessing his treatment response has been symptom‐driven. Posttreatment, he has had some improvement in his neurological function and has not suffered any repeat ischemic events.

One additional tool used to assess our patient’s treatment response was the ClonoSEQ MRD testing, which was negative after 6 cycles of R‐CHOP. MRD testing using ctDNA is a relatively new entity and can aid in assessing treatment response and detecting early relapse in diffuse large B‐cell lymphoma (DLBCL) [12]. Specifically, studies have shown that downtrending ctDNA levels throughout a patient’s treatment course are associated with better outcomes [13]. Additionally, MRD testing can also detect disease relapse prior to evidence of relapse on surveillance imaging [14, 15]. For our patient, who had minimal evidence of disease on imaging, this strategy of disease surveillance in conjunction with clinical assessment of symptoms will likely be the mainstay of assessing disease status for him.

## 4. Conclusion

In the above case, we presented a patient who was diagnosed with IVLBCL as a cause of cryptogenic strokes. This patient’s presentation was complicated by the lack of clear imaging findings and an inability to clearly track his response to treatment. This case demonstrates the importance of a thorough workup of underlying causes of cryptogenic stroke as well as using novel methods such as ctDNA testing to determine disease status.

## Funding

No funding was received for this manuscript.

## Disclosure

All authors have read and approved the final version of the manuscript. Dr. Veena Krishnan had full access to all of the data in this study and takes complete responsibility for the integrity of the data and the accuracy of the data analysis.

## Conflicts of Interest

The authors declare no conflicts of interest.

## Supporting Information

Additional supporting information can be found online in the Supporting Information section.

## Supporting information


**Supporting Information** Case report checklist (9959762).

## Data Availability

Data sharing is not applicable to this article as no datasets were generated or analyzed during the current study.
